# High level of treatment failure and drug resistance to first-line antiretroviral therapies among HIV-infected children receiving decentralized care in Senegal

**DOI:** 10.1186/s12887-019-1420-z

**Published:** 2019-02-05

**Authors:** Abdoul-Magib Cissé, Gabrièle Laborde-Balen, Khady Kébé-Fall, Aboubacry Dramé, Halimatou Diop, Karim Diop, FatouNiasse-Traoré, Mohamed Coulibaly, Ndeye-Ngone Have, Nicole Vidal, Safiatou Thiam, Abdoulaye S. Wade, Martine Peeters, Bernard Taverne, Philippe Msellati, Coumba Touré-Kane

**Affiliations:** 1 Service de pédiatrie Établissement Public de Santé (EPS) de Mbour, UFR Sciences de la Santé, Thiès University, Thiès, Senegal; 2 Expertise France, Paris, France; 3 ANRS-Senegal site, Dakar, Senegal; 4 Centre régional de recherche et de formation à la prise en charge clinique de Fann (CRCF), Dakar, Senegal; 5 Bacteriology-Virology Laboratory, A. Le Dantec CHU, Cheikh Anta Diop University, Dakar, Senegal; 6 Division de lutte contre le sidxa et les IST (DLSI), Ministry of Health and Social Action (MSAS), Dakar, Senegal; 7 Conseil national de lutte contre le sida (CNLS), Dakar, Senegal; 8 Réseau national des associations de personnes vivant avec le VIH au (RNP+), Dakar, Senegal; 9 Institut de Recherche pour le Développement (IRD), Montpellier, France; 10 TransVIHMI, IRD, PACCI, 18 BP 1954, Abidjan 18, Abidjan, Côte d’Ivoire

**Keywords:** Pediatric HIV, Antiretroviral, Viral resistance, Decentralization, Senegal

## Abstract

**Background:**

In Senegal in 2015, an estimated 4800 children were living with HIV, with 1200 receiving ARV treatment, of whom half had follow-up care in decentralized sites outside Dakar.

However, until now no studies have determined the efficacy of pediatric treatment in decentralized settings, even though the emergence of viral resistance, particularly among children in Africa, is a well-known phenomenon. This study aimed to assess the virological status of HIV-infected children in all decentralized facilities to help improve access to quality care.

**Methods:**

A cross-sectional epidemiological and virological study was conducted in all of Senegal’s regions, except Dakar, between March and June 2015 and sought to include all HIV-infected children and adolescents (0–19 years), treated or not with ARVs.

Socio-demographic and clinical data and a blood sample on blotting paper were collected for children from treatment sites. Samples were routed on public transportation, assisted by a network of community health workers. A viral load (VL) assay was performed for each child, followed by genotyping when it exceeded 1000 copies/mL (3 log_10_).

**Results:**

Of the 851 identified children, 666 (78%) were enrolled in the study. Half of the children were girls, and the average age was 8 years (6 months–19 years). Most of the children (96.7%) were infected with HIV-1, and 90% were treated with ART, primarily with AZT + 3TC + NVP/EFV therapeutic regimen. The median duration of time on ART was 21 months (1–129). VL was measured for 2% of children before this study. Almost two-thirds (64%) of the children are experiencing virological failure. Among them, there was resistance to at least one drug for 86.5% of cases. Also, 25% children presented resistance to one drug and 40% to two out of three. For nearly one-third of the children presenting resistance, none of the three drugs of the treatment was active. Factors associated with virological failure were male sex, follow-up by a generalist rather than a specialist, and treatment interruptions.

**Conclusions:**

We observed a high level of virological failure and a high percentage of viral resistance among children receiving health care in decentralized facilities in Senegal.

## Background

The use of antiretroviral therapy (ART) has considerably reduced morbidity and mortality among HIV-infected children. In 2013, WHO recommended universal treatment for all children up to age 10 years in 2015 [1]. Globally, ART coverage has increased from 21% in 2010 to 49% in 2015, for 1.8 million children infected with HIV [2].

However, although ART is a positive treatment option, high levels of resistance persist [3]. Some authors even speak about a 4th phase of the HIV pandemic [4]. Pretreatment drugs resistances are already at a dramatic level, beyond 10% in Southern and East Africa in 2016 [5]. Children, more so than adults, are particularly vulnerable to resistance, especially in resource-limited countries, due to a frequently high viral load (VL) before the start of treatment, unavailability of adapted drug formulations, frequent poor adherence, and limited VL access [6–9].

In sub-Saharan Africa, levels of virological failure, with or without resistance mutations in children, range between 13 and 56%, depending on the studies and countries [10–13]. Senegal is a country with low HIV prevalence—0.5% in the adult population. In 2015, an estimated 4800 children were living with HIV in Senegal, of whom only 25% had access to ART, compared to 42% for adults [14]. These children received follow-up care either in reference centers in Dakar or in sites located throughout the country [15].

Senegal’s health pyramid includes health centers and level-I–III hospitals that correspond to departmental, regional, and national reference levels, respectively.

Pediatric HIV care is well-structured in the capital’s reference health facilities, with services for children living with HIV in some hospitals. In regions outside Dakar, the quality of care provision for children often suffers from poor access to VL and care providers’ lack of experience in pediatric HIV. This first country-wide study was conducted in 2015 to assess the quality of decentralized care for children living with HIV using biological markers (viral load, genotyping).

The study also aimed to detect virological failures in order to change the therapeutic regime in the event of resistance, and thus help improve the quality of care for each child. Finally, this research tested a nationwide sample delivery circuit using dried blood spot (DBS) to facilitate access to viral load in isolated locations.

## Methods

This cross-sectional study, implemented throughout the country between March and June 2015, sought to include all HIV-infected children and adolescents (0–19 years), whether treated with ART or not, and receiving follow-up care in decentralized structures. Inclusion criteria were: to be HIV infected, to be 0–19 years old, to be followed for care out of the Dakar region and parents consenting to the study. They were no exclusion criteria.

The physician and team in each site conducted the recruitment process. All sites, outside of Dakar region, caring for HIV-infected children (72 in 2015) were involved in the study. Biological (viral load, genotyping), socio-demographic, and clinical data were collected.

In addition to collecting socio-demographic and clinical data for each child, a DBS blood sample [16] was performed and routed on public transportation to the reference laboratory, with assistance from a network of community health workers from associations.

After extraction of nucleic acids using the NucliSENSEasyQ™ technique, VL was quantified (Abbott Real Time HIV-1 m2000rt quantitative assay™ Abbott Laboratories, Chicago, IL). A serological confirmation was performed by Determine™ and BiSpot™ for all undetectable viral loads. Genotyping was performed according to the ANRS protocol for any VL greater than or equal to 1000 copies/mL (≥3 log_10_) for children treated with ART for at least six months. The sequences were submitted online to the Stanford database (https://hivdb.stanford.edu/) and analyzed using the ANRS algorithm, version 2015 (hivdb.stanford.edu/downloads/ANRS.xml). Phylogenetic analyzes were performed to identify HIV-1 subtypes [17].

Genotyping was conducted either at the bacteriology-virology laboratory (BVL) in Dakar (Senegal) or at the IRD UMI 233-TransVIHMI laboratory in Montpellier (France), two reference virology laboratories. Epidemiological analyses were performed using Stata release 13® software (2013). Statistical analysis was performed using Chi2 tests and a logistic regression model. A threshold of 0.20 was chosen to select variables in univariate analysis and to determine whether they would be considered in multi-variate models. The overall threshold for testing was 5%.

The protocol was approved by the National Ethics Committee for Research in Health in Senegal on 16 December 2014 under no. 377/MSAS/DPRS/CNERS. All parents or guardians provided written informed consent.

## Results

Of the 851 HIV-infected children identified by physicians, 672 were recruited, which represented 78.9% of the previously identified children. Some 21% of the children were not included for various reasons, including travel at the time of the survey and difficulties reaching the family.

All 672 children had a DBS sample, and 666 were enrolled in the study. Three samples were performed improperly, and three children were misdiagnosed as HIV-infected.

### Characteristics of the study population

The 666 children’s main characteristics were (Table 1).

**Table 1:** Characteristics of the 666 children and adolescents enrolled in the EnPRISE study, 2015, Senegal

*Characteristics*	*n*	*n*	*%*	*%*
*N*	666			
*Gender (n = 666)*				
Girl	328		49%	
*Age (n = 666)*				
< 5 years	159		24%	
5–10 years	253		38%	
10–14 years	213		32%	
> 14 years	41		6%	
*Education (n = 666)*				
No formal education	235		35%	
Educated	431		65%	
Primary		255		59%
Arabic-Koranic^a^		99		23%
Secondary		47		11%
Unavailable		30		7%
*Family status (n = 666)*				
Both parents alive	273		41%	
One parent alive	292		44%	
Orphans of both parents	101		15%	
*Testing of siblings (n = 666)*				
No siblings	82		13%	
Siblings	485		72%	
No one tested	135		28%	
At least one tested	140		29%	
All tested	210		43%	
Unavailable	99		15%	
*Type of facility* ^*b*^ *(n = 666)*				
Health Center	322		48%	
Level-I Hospital	141		21%	
Level-II Hospital	196		30%	
Level-III Hospital	7		1%	
*Responsible for treatment (n = 666)*				
General practitioner	432		65%	
Pediatrician	234		35%	
*Disclosure (n = 666)*				
Children < 8 years (not notified)	243		37%	
Age ≥ 8 years	300		45%	
Notified of status	42		14%	
Not notified of status	258		86%	
Unavailable	123		18%	
*Gateway to care* ^*c*^ *(n = 666)*				
Care	347		52%	
Familial	270		40%	
PMTCT	12		2%	
Unavailable	37		6%	
*Regularity of follow-up* ^*d*^ *(n = 666)*				
Regular	482		72%	
Not regular	128		19%	
Unavailable	56		9%	
*Prolonged treatment interruptions* ^*e*^ *(n=666)*				
Yes	75		11%	
No	586		88%	
Unavailable	5		1%	
*Nutritional status* ^*f*^ *(n = 666)*				
Normal	393		59%	
Moderate acute malnutrition (MAM)	95		14%	
Severe acute malnutrition (SAM)	87		13%	
Overweight	28		4%	
Unavailable	63		10%	
*Exposure to PMTCT (n = 666)*				
Yes	45		7%	
No	468		70%	
Age < 10 years	412		88%	
Age ≥ 10 years	56		12%	
Unavailable	153		23%	
*ART Therapy (n = 666)*				
Children untreated	65		10%	
Children treated	601		90%	
*Viral load (n = 666)*				
Treatment ≤6 months	103		17%	
Treatment > 6 months	498		83%	
< 1000 cp/mL		178		36%
≥1000 cp/mL		320		64%
*Duration of follow-up (treatment > 6 months n = 498)*				
6–12 months	110		22%	
13–24 months	113		23%	
> 24 months	275		55%	
*Treatment regimen (treatment > 6 months n = 498)* ^*g*^				
AZT + 3TC + NVP/EFV	425		85%	
Other NRTI+NNRTI combinations	37		8%	
Combination with PI	36		7%	
*Adherence* ^*h*^ *(all children on treatment n = 601)*				
Good	372		61%	
Average	81		13%	
Poor	61		10%	
Unavailable	87		14%	

^a^Franco-Arabic/Koranic schools based on the study of the Koran and Arabic language

^b^Level-I Hospital, departmental level; Level-II Hospital, regional level; Level-III Hospital, national level

^c^Gateway to care: place of first consultation leading to HIV testing

^d^Regularity of follow-up: quarterly

^e^Prolonged treatment interruptions: greater than one month

^f^Nutritional status: based on WHO criteria

^g^Treatment regimen

^h^Adherence: estimated by physician

The sex ratio was 1.03. The average age was 8 years (range 6 months–19 years) with IQR 5 and 12. Eight percent of the children older than 7 years were not enrolled in school. Among the enrolled children, 59% were in primary school, 11% in secondary, and 23% were in a Koranic/Franco-Arabic school.

Overall, 64% of the children lived with one of their parents, mainly their mothers. Forty-four percent of the children had a deceased father or mother, and 15% were double orphans. The families had 2.6 other children on average, of whom 1.3 had been tested. Only 43% of families had tested all siblings. When testing was done, the average number of other infected children per family was 1. The percentage for HIV infection from an index case in a family was 33%.

The children received follow-up in treatment sites with widely varying characteristics depending on the level of decentralization. Each of the thirteen regions outside of Dakar has a regional hospital that usually includes a pediatric unit, located in the region’s capital. Health centers in villages or medium-sized cities are headed by general practitioners. The number of HIV-infected children receiving follow-up is usually low. Only 10% of the sites provided follow-up for more than 20 HIV-infected children, and 68% provided follow-up for less than 10 children. The average number of children receiving care was nine per site (1–38).

### Clinical and treatment data

Children (96.7%) were mostly infected by HIV-1, 2.2% by HIV-2, and 0.9% by dual infection. The serotype could not be determined for one child.

The median duration of follow-up was 28 months (10–51). Mean age at time of testing was 5.5 years (2 months–16 years). Circumstances for discovering infection were care (consultations and hospitalizations) for 52% of cases and family testing for 40% of cases. Failure of PMTCT (prevention of mother-to-child transmission) accounted for 2%. The most commonly used method for diagnosis was retroviral serology, with only 8 children tested by PCR. Among the 543 children for whom information was available, only 14% of children aged 8 years and older were fully notified of their serological status. According to the physicians’ estimation, 72% of the children came regularly to their medical appointments, 61% had good adherence, but 11% have suffered one or more prolonged treatment interruptions, lasting more than one month. Only 8.7% of the 513 children with available data had access to PMTCT. Among the 468 children who did not receive PMTCT services, 88% were under 10 years old. According to WHO standards (weight/height index or mid-upper arm circumference), nutritional status was normal for 59% of the children. Over one-fourth of the children (27%) suffered from malnutrition, including 13% with severe acute malnutrition (SAM) and 14% with moderate acute malnutrition (MAM). Four percent of the children were overweight. At the time of the study, 85% of the children were asymptomatic. Dermatosis, respiratory infections, candidiasis, and diarrhea were the most frequent signs for symptomatic children.

Ninety percent of the children (601/666) received ART, including 498 for at least six months: 110 had been treated for 6 to 12 months; 113 for 13 to 24 months; and 275 for more than 24 months. The median follow-up under treatment was 21 months (1–129). Ninety-six percent of children treated for at least six months were on first-line regimens, mainly AZT + 3TC + NVP/EFV (507 children treated with NVP and 64 children treated with EFV) and 4% were on second-line regimens, mainly ABC + 3TC + LPV/r.

### Virological data and resistance tests

During their follow-up, 64% of the children had at least one CD4+ count, 21% had two or more, and 2% had at least one documented VL measurement before this study.

In this study, VL results showed that only 26% of children treated for at least six months had an undetectable VL. The percentage of children with a detectable VL < 3 log_10_ (1000 copies/mL) was 10%. The virological failure (VL > 1000 copies/mL) rate was 64% (320 children). The median VL was 4 log_10_ [3 log_10_–5.7 log_10_]. Ten percent of children had a VL greater than 5 log_10_ (Table 2). All children with virological failure were tested for resistance (genotyping). For technical reasons, 16 analyses could not be performed, including 3 for children infected by HIV-2 and 13 because of problems with sequence quality. Thus, 304 (95%) results were reported.

**Table 2:** Viral load and resistance profiles of children with virological failure (VL > 1000 copies/mL)

Variables	n/N tested	%	%
*Viral load (VL)*			
VL ≥ 3 log_10_	320/498	64	
3 log_10_ ≤ VL ≤ 3.7 log_10_	120/320		37.5
3.7 log_10_ < VL ≤ 4 log_10_	40/320		12.5
4 log_10_ < VL ≤ 4.7 log_10_	100/320		31.25
4.7 log_10_ < VL ≤ 5 log_10_	27/320		8.44
5 > VL log_10_	33/320		10.31
Median VL = 4 log_10_			
*Genotypes performed*	304/320		95
*Frequency of drug-resistant virus*			
Resistance to at least one ART	263/304	86.5	
Resistance to NRTIs only	5/263		1.9
Resistance to NNRTIs only	60/263		22.8
Resistance to NRTIs+NNRTIs	197/263		74.9
Resistance to NRTIs+NNRTIs+PIs	1/263		0.4
*Frequency of drug resistance to the different drugs*			
AZT	90/263		34.22
d4T	93/263		35.36
3TC/FTC	195/263		74.14
ABC	165/263		62.73
ddI	18/263		6.84
TDF	43/263		16.34
EFV	238/263		90.49
NVP	253/263		96.19
ETR^a^	125/263		47.52
RPV^a^	136/263		51.71
*Predicted resistance to the current regimen (3 drugs)*			
No resistance	5/263		1.9
1 out of 3 drugs	66/263		25.09
2 out of 3 drugs	106/263		40.3
3 out of 3 drugs	86/263		32.69

^a^Drug not given in the current ART regimen

Of the 304 children with virological failure and a genotyping result, 263 (86.5%) had resistance to at least one drug (Table 2). Nearly 75 % (74.9%) of children who had at least one resistance were resistant to both an NRTI and an NNRTI. Five children (1.9%) presented resistance only to NRTIs, and 60 children (22.8%) were resistant to only NNRTIs. The highest resistance rates were observed for NVP (96%), EFV (90%), and 3TC/FTC (74%)—drugs in the current treatment. These NNRTIs included resistance to both EFV and NVP in some children, even when only one of the two drugs was part of their treatment. High levels of cross-resistance were observed—for example for ABC (62%), taken by only 4% of the children, and also for ETR (47%) and RPV (51%), that were not in any regimen (Table 2). For TDF, 16% of the children who were tested presented resistance, though only 41/601 (7%) had taken it. For resistance due to current treatment, only 2% of the children with a genotype test result were sensitive to all three drugs in their treatment, 25% had resistance to one drug, and 40% to two out of the three. Among the 263 children with resistance, for 86 of them (32.69%), none of the three treatment drugs were active. The predominant resistance mutation, of those related to NRTIs, was M184 V, linked to the use of 3TC/FTC (73%). The K103 N mutation was the most common (48%) among mutations related to NNRTIs. The presence of thymidine analogue mutations (TAMs) (33%) was also observed (see Table 2). In 22% of cases, M184 V was associated with at least two TAMs.

Phylogenetic analysis showed results consistent with the genetic diversity present in the general population in Senegal. Recombinant form CRF02_AG predominated at 71.05%.

### Factors associated with a high viral load

The factors associated with virological failure in a logistic regression were male sex (*p* = 0.023), follow-up care by a generalist rather than a pediatrician (0.036), and prolonged treatment interruptions (0.001). No significant difference was found in terms of mother’s vital status, parents’ marital status, existence of an HIV-positive sibling, declared compliance, or regularity of follow-up (see Table 3).

**Table 3:** Factors associated with high viral load, EnPRISE, Senegal, 2015

*Character-istics*	*Total*	*VL < 1000*	*VL ≥ 1000*	*p value*	*Odds ratio*	*p*
*Gender*						
Male	338/666 (51%)	107/241	228/417	0.011	0.69 (0.50–0.95)	0.023
*Marital status*						
Mother in partnership	384/516	129/186	255/330	0.048	0.67 (0.44–1.01)	0.056
*Health professional*						
Paedia-trician	233/657	99/241	134/416	0.022	1.43 (1.02–2.0)	0.036
*Regular monitoring*						
No	128/610	29/223	99/387	< 0.001	1.08 (0.5–2.33)	
*Anthro-pometrics*						
WAZ < −3	66/420	18/162	48/258	0.040*		
HAZ < −3	98/596	25/215	73/381	0.017*		
*Compliance*						
Bad	142/514	38/192	104/322	0.002	1.62(0.94–4.93)	0.085
*Prolonged ARV interruption*						
Yes	75/661	14/242	61/419	0.001	2.77 (1.51–5.09)	0.001

*Excluded from the multivariate analysis. Too many data missing

## Discussion

Sixty-four percent of children receiving follow-up in decentralized settings in Senegal in 2015 had virological failure, and of the 304 children tested for resistance, 263 (86.5%) presented resistance to at least one antiretroviral drug. This study is the first national assessment in Senegal of the virological status of HIV-infected children who are receiving care outside of reference centers located in Dakar. Its interest lies in the large number of children included and its reach across the entire country. All health facilities that treat HIV-infected children throughout Senegal, with a wide range of technical platforms and economic and geographic environments, were included. Resistance tests were performed for 95% of the children with high VL, which provides a good estimation of their virological situation. Therefore, this study accurately reflects decentralized treatment for children in Senegal. The collected data were transmitted to each site’s physicians so that they could adapt each child’s antiretroviral treatment based on the biological results.

Nevertheless, there are limitations: 21% of the children were not included for various reasons, including travel at time of the survey and difficulties reaching the family. Moreover, this is a cross-sectional study, with a single sample. The reliability of this type of survey is weak and factors associated with treatment failure should be interpreted cautiously. Also, since there were many sites and a very small number of children in some of them, we were not able to analyze the factors due to site effect.

Compliance data were based solely on physicians’ estimates, with some subjectivity. Socio-economic factors related to therapeutic failure have not been explored. Missing data on some variables must also be considered.

In this study, percentage of observed PMTCT failure (2%) is close to the 3.1% transmission rate for the entire country in 2015 [15]. However, the number of children who received PMTCT interventions is low. The majority of them who were not exposed to PMTCT were under 10 years, and, therefore, were infected after 2007, when PMTCT was already being decentralized throughout the country.

Coverage in Senegal is relatively low, with only 36% of women having received ART during PMTCT in 2015. This rate is close to that of some neighboring countries, such as Mali (33%) or Niger (28%), but lower than others, such as Burkina Faso (79%) or Côte d’Ivoire (89%) [14].

Along with expanding PMTCT coverage, testing strategies for children outside of these programs must be strengthened. The study shows that testing for children remains low in affected families, as evidenced by the high percentage of siblings of HIV-infected children who were not tested. In addition, the number of children receiving care is low compared to the number of adult PLHIV who do, since it amounts to less than 7% of infected people treated by ART nationwide; this exemplifies one of the consequences of limited testing [14]. While barriers to testing children in Africa are well known, routine family testing, particularly for siblings of infected children, with the support of PLHIV associations seems to be the most efficient strategy to improve early diagnosis and treatment [18, 19].

This study reveals that nearly two-thirds of children treated by ART and receiving care in decentralized sites are experiencing treatment failure, according to the 2013 WHO standards. Various studies have shown high rates for virological failure in children treated with ART in resource-limited countries. Meta-analyses of countries in Africa, Asia, the Caribbean, and Latin America have reported average treatment failure rates from 26 to 36% with wide variations (13 to 71%) depending on the countries, the type of study, treatment duration, and VL thresholds used to define virological failure [6, 7, 10]. In Africa, rates vary but may be high, depending on the contexts, with 17% in a reference center in Cameroon [20] and 25% in a rural setting in Tanzania [13]. Two cross-sectional studies showed failure rates of 51.6% in a rural and semi-urban setting in Togo [12] and 60% in a hospital in the capital of Central African Republic [21]. In Senegal, the only available study, conducted in 2010 with children receiving care in a hospital in the capital, showed a failure rate of 56% after 20 months of ART [11].

Some factors associated with virological failure—poor adherence, prolonged treatment interruption, and irregular follow-up—are known [6, 13, 20]. There is less consensus regarding other factors. Male sex, associated with failure in our study, is not associated in other studies [13]; status of orphan, which does not appear to be decisive here, is relevant elsewhere [20]. The high percentage of virological failure in the smallest facilities and when treatment services are headed by a general practitioner rather than a pediatrician raises further questions. This could be explained by lack of equipment as well as limited experience and practice among the physicians who provide care for a small number of children. This finding echoes the higher mortality described for children in Zambia in rural facilities compared to those in urban settings [22].

Most children with virological failure show resistance to one or two classes of antiretrovirals. Resistance, particularly to NNRTIs, is high and remains stable over time. For nearly one-third of the children presenting resistance, no drug is active, and 40% are only sensitive to one drug. The rate of cross-resistance is concerning. Accumulated resistance mutations not only make children resistant to their treatment, but also create resistance to alternative drugs.

Similar results are found in urban settings in Senegal [11], as well as in other countries in West, Central, and East Africa [6, 12, 21]. Exposure to ART during PMTCT, noted in some studies [6], was unable to explain the occurrence of resistance here, since less than 10% of infected children received prophylaxis. A study in India also found a low level of transmitted resistance (5.7%) for HIV sub-type C despite high levels of VL [23]. In Peru, it was shown that brief exposure to ART through PMTCT did not have a significant impact on the outcome for infected children [24].

The properties of drugs (low genetic barrier), difficult adherence for children, young children’s poorer response to ART, frequently high VLs at treatment initiation, and administration of sub-therapeutic doses are known factors of failure [6]. Non-existent virological monitoring during the decentralization process contributes to late diagnosis of failure [8]. Children with virological failure must be changed to treatment regimens that include protease inhibitors, but first-line therapies with higher genetic barriers should also be considered.

Fortunately, the situation is reversible. Through measures implemented with support from research and public health programs, the Albert Royer Children’s Hospital in Dakar managed to invert the proportions of viral suppression, from 34 to 80% between 2010 and 2016 [25]. Thus, it is possible, through targeted interventions [26], to strengthen care for children and improve the situation in decentralized sites even if these interventions are adapted to different contexts.

Routine virological monitoring is rarely done in the decentralized sites. While 90% of children are treated with ART, only 2% have had VL tested at least once, and measuring VL has only been recently included in both international and national recommendations.

For most of the children, this study was an opportunity to have their first virological test. It helped diagnose treatment failures, which are more difficult to detect using clinical and immunological criteria [13, 27]. Though now highly recommended by WHO, regularly measuring VL outside of Dakar runs up against a lack of equipment and reagents and weaknesses in the current circuit for routing samples to laboratories for biological analyses.

This study has shown the feasibility of virological monitoring of HIV-infected children, living throughout Senegal, by implementing a DBS-collection and -routing circuit. This circuit, supported by a network of community health workers and public transportation, has proven to be flexible, effective, and inexpensive. It is easily reproducible by the national AIDS control program, but its sustainability depends on funding for the circuit, the availability of reagents, its continuous coordination, and rapid reporting of results.

The problem of therapeutic failures and viral resistance has become a major international issue for children and adults alike. In 2017, a WHO report noted the rapid development of viral resistance to ARVs. Its 2017–2021 action plan recommends widespread virological follow-up, switching treatment regimens, better support systems for adherence, and resistance surveillance at country level [28, 29]. The implementation of these recommendations in developing countries requires the joint commitment of States and the international community.

## Conclusion

This study is the first of its type conducted in the regions of Senegal. The high number of children in treatment failure in decentralized facilities shows the limited efficacy of care services, a situation also found in other resource-limited countries. However, improvement is possible through targeted measures. This involves overall strengthening of facilities, managing notifications, improvements in children’s social and economic environment such as community people involvement, social support for families (food, schooling, etc.), support for adherence, and a system for regular virological monitoring. It also requires adjusting treatments quickly for children with virological failure, by introducing protease inhibitors, considering first-line therapies with high genetic barriers, and lastly, ensuring the availability of third-line ART. Implementing these measures is crucial if we hope to achieve the UNAIDS 90–90-90 goals and eradicate the epidemic by 2030 [30].

## Abbreviations

3TC: Lamivudine
ABC: Abacavir
AIDS: Acquired Immunodeficiency Syndrome
ANRS: Agence Nationale de Recherches sur le SIDA et les hépatites virales
ART: Antiretroviral Therapy
ARV: Antiretroviral
AZT: Zidovudine
BVL: Bacterio-Virology Laboratory
CD4 +: Cluster Differentiation 4 +.
CRF02-AG: Circulating Recombinant Form AG-02
DBS: Dried Blood Spot
EFV: Efavirenz
ETR: Etravirine
FTC: Emtricitabine
HIV: Human Immunodeficiency Virus
IQR: Inter Quartiles Range
IRD: Institut de Recherche pour le Développement
LPN/r: Lopinavir boosted by ritonavir
MAM: Moderate Acute Malnutrition
NNRTI: Non-Nucleoside Reverse Transcriptase Inhibitor
NRTI: Nucleoside Reverse Transcriptase Inhibitor
NVP: Nevirapine
PCR: Polymerase Chain Reaction
PLHIV: People Living with HIV
PMTCT: Prevention of Mother to Child Transmission
RPV: Rilpivirine
SAM: Severe Acute Malnutrition
TAM: Thymidine Analogues Mutations
TDF: Tenofovir
UMI: Unité Mixte Internationale (International Joint Unit)
UNAIDS: Joint United Nations Programme on HIV/AIDS
VL: Viral Load
WHO: World Health Organization
